# Undergraduates’ achievement and satisfaction: the role of study-related factors and soft skills

**DOI:** 10.3389/fpsyg.2025.1653072

**Published:** 2025-10-08

**Authors:** Adi Sharabi, Barbara Carretti, Marisol Cueli, Celestino Rodríguez, Gerardo Pellegrino

**Affiliations:** ^1^Faculty of Education, Kibbutzim College of Education Technology, and the Arts, Tel Aviv, Israel; ^2^Department of General Psychology, University of Padova, Padova, Italy; ^3^Department of Psychology, University of Oviedo, Oviedo, Spain

**Keywords:** academic self-efficacy, academic satisfaction, self-regulated learning, soft skills, higher education

## Abstract

The present study tested a comprehensive model in which five social, emotional, and behavioral (SEB) skill domains (self-management, social engagement, cooperation, emotional resilience, and innovation; also known as “soft skills”) were associated with academic achievement and satisfaction directly and through the mediation of academic self-efficacy and self-regulated learning (SRL) strategies. The participants were 319 undergraduate students (mean age = 22.74, SD = 4.68; 255 females) from Spain, Italy, and Israel. Self-report questionnaires were administered for this study. Our results showed that academic self-efficacy and SRL significantly mediated the relationships between self-management and social engagement skills with academic achievement, after controlling for sex, age, year of study, and nationality. Academic self-efficacy uniquely mediated the relationship between SEB skills and academic achievement and satisfaction. Fostering socio-emotional skills can benefit students during higher education and lead to positive perceptions of themselves as competent and self-regulated learners, which can lead to greater academic satisfaction and achievement.

## 1 Introduction

Decades of research on academic achievement and satisfaction have sought to identify the individual differences that predict these outcomes and develop tools and strategies to help higher education students reach their educational goals and succeed in their studies (Ben-Eliyahu, 2019; Kryshko et al., 2023). Academic learning is a complex phenomenon, and students must rely on a range of personal characteristics, including cognitive abilities, study strategies, and motivational beliefs, to overcome learning challenges (Casali and Meneghetti, 2023; Mega et al., 2014; Richardson et al., 2012; Russell et al., 2022).

In recent years, more attention has been given to the role of general soft skills and personal competencies that support students in approaching complex challenges, not only during their studies but also in their future careers (Casali et al., 2024; Park et al., 2017; Soto et al., 2022b). These skills have been given various names, such as soft skills, life skills, character skills, or social–emotional skills (Napolitano et al., 2021). Their importance in the educational context has been widely recognized by various national and international institutions, including the World Economic Forum (2016) and the OECD (2015, 2021). However, research on skills and competencies has been hindered by the presence of multiple theoretical frameworks and the lack of well-defined instruments to measure them (Duckworth and Yeager, 2015; Napolitano et al., 2021). Moreover, to date, only a few studies have examined the contribution of skills and competencies alongside core motivational and metacognitive factors, such as study strategies and academic self-efficacy (Casali and Meneghetti, 2023; Feraco et al., 2022a, 2025).

Therefore, the present study aimed to examine the relations between five skill domains derived from the recent Social, Emotional, and Behavioral (SEB) skills framework (Soto et al., 2022a), academic self-efficacy and self-regulated learning (SRL) strategies (Bandura, 2012; Casali and Meneghetti, 2023), and their role as predictors of academic achievement (in terms of average grades), and academic satisfaction (i.e., students’ subjective evaluation of the quality of their university lives; Casali et al., 2024; Huebner et al., 2012). The study aims to provide a more comprehensive view of learning in higher education and highlight how SEB skills, along with other study-related factors, contribute to student achievement and satisfaction.

## 2 Theoretical background

### 2.1 What leads to academic success?

Contemporary models of successful academic learning (Ben-Eliyahu, 2019; Collie and Martin, 2024; Wang et al., 2019) emphasize the role of individual characteristics in promoting students’ learning and adjustment. In particular, motivational and metacognitive factors (such as academic self-efficacy, intrinsic motivation, goal orientation, and SRL) have been widely studied as key antecedents of students’ academic achievement, satisfaction, and well-being (Ben-Eliyahu, 2019; Casali and Meneghetti, 2023; Feraco et al., 2022a,b; Richardson et al., 2012).

Among these factors, academic self-efficacy and self-regulated learning (SRL) have received particular attention (Casali et al., 2024; Mega et al., 2014). SRL is a multidimensional construct that emphasizes the active role of the learners and their control over the use of learning strategies (Mega et al., 2014; Panadero, 2017; Theobald, 2021; Zimmerman, 2000). Self-regulated students are aware of task requirements and their own needs to optimize their learning experience. They are aware of the strategies that can lead to optimal learning and know how to apply them to enhance their performance (Mega et al., 2014; Theobald, 2021). SRL is associated with other important study-related factors, such as learning goals, theories of intelligence, and academic self-efficacy (Casali et al., 2022; Mega et al., 2014). Ultimately, students who deploy better SRL strategies also obtain higher grades (Casali et al., 2022; Richardson et al., 2012).

Self-efficacy refers to people’s beliefs regarding their ability to meet desired outcomes, influence events related to their lives, or achieve goals (Bandura, 1982, 2012). Academic self-efficacy reflects an individual’s confidence in their ability to succeed academically and meet their learning requirements (Bandura, 1982, 2018; García-Ros et al., 2023; Mega et al., 2014). Various studies, including meta-analytic research, have shown that academic self-efficacy is strongly associated with academic achievement (Al-Abyadh and Abdel Azeem, 2022; Basileo et al., 2024; Høigaard et al., 2015; Richardson et al., 2012), such that students who believe they can succeed in their studies are also the ones who achieve academic success. Academic self-efficacy seems to be important not only for academic achievement, but also for students’ psychological well-being (Casali et al., 2022, 2024; Feraco et al., 2022a,b; Sharabi et al., 2016; Sharabi, 2024). In particular, academic self-efficacy is positively associated with academic satisfaction (Casali et al., 2024): Students who feel more capable of achieving their academic goals are also more satisfied with their academic path.

While much of the existing research has focused on these study-related factors, recent approaches call for a broader view of what supports students’ success. In particular, general life skills—often referred to as soft skills—are gaining attention as complementary resources that can influence both academic and non-academic outcomes (Collie and Martin, 2024; Soto et al., 2022b). These skills may interact with traditional motivational and metacognitive factors to shape how students cope with challenges and pursue their goals during university (Casali and Meneghetti, 2023). The present study builds on this perspective, examining how soft skills contribute to student success alongside well-established study-related factors.

### 2.2 Soft skills and educational outcomes

Recently, greater attention has been given to the role of general individual skills on educational outcomes. Multiple theoretical frameworks consider personal competencies as resources that support students in navigating learning tasks (Ben-Eliyahu, 2019; Collie and Martin, 2024; Wang et al., 2019). For example, the integrated Self-Regulated Learning (iSRL) model (Ben-Eliyahu, 2019) includes personal skills, along with classical motivational and metacognitive factors, among the individual factors that promote learning achievement. The Integrative Development-in-Sociocultural Context Model (Wang et al., 2019) and the Social–Emotional Flourishing Framework (Collie and Martin, 2024) also posit that social, emotional, and cognitive skills are fundamental personal antecedents of students’ motivation, engagement, and educational outcomes.

Previous empirical works (Casali et al., 2024; Feraco et al., 2022a,b) have used the term soft skills to describe the malleable competencies that enable people to interact effectively with others and achieve their personal goals (Feraco et al., 2022a; Robles, 2012). These skills represent an individual’s capacity to respond appropriately in diverse situations in ways that align with the demands of the situation (Soto et al., 2022a). Feraco et al. (2022a,b) have explored the associations between soft skills and educational outcomes in secondary school students. They observed that six soft skills derived from the World Economic Forum (2016) framework—adaptability, curiosity, perseverance, social awareness, initiative, and leadership—can be combined into a unique “soft skill” second-order factor that is positively associated with SRL strategies, academic motivation, general life satisfaction, and positive emotions towards school. The authors observed that the contribution of soft skills to academic achievement may not be direct but mediated by SRL, academic motivation, and emotions toward school. Indeed, soft skills are considered domain-general abilities (i.e., abilities relevant across multiple life contexts) that, although not specific to educational settings, can support domain-specific factors (e.g., motivational and metacognitive processes) in school and higher education. In another study, soft skills were indirectly related to arithmetic performance through the mediation of achievement emotions and academic motivation, suggesting that these skills may be important not only for general achievement but also for specific subjects (Feraco et al., 2024b). Moving to the higher education context, Casali et al. (2024) explored the contribution of five soft skills (i.e., creativity, critical thinking, curiosity, perseverance, and social intelligence) to university students’ achievement, and academic and life satisfaction, showing that both critical thinking and curiosity were positively associated with academic satisfaction, even after accounting for other study-related factors (i.e., academic self-efficacy, SRL strategies, learning goals).

These preliminary studies suggest that skills and competencies can play a role in students’ achievements and satisfaction. More interestingly, the association between soft skills and student outcomes may not be direct but mediated by motivational and metacognitive factors (Casali and Meneghetti, 2023; Feraco et al., 2022a,b). These skills can help students engage positively in learning activities and find functional strategies to cope with academic demands, which in turn has a positive impact on their achievement and satisfaction. However, a main limitation of these studies is that they have focused on specific skills without considering a more comprehensive theoretical framework of soft skills.

### 2.3 Social, emotional, and behavioral skills: a new theoretical framework for soft skills

Research on the role of skills and competences in the educational context is characterized by the presence of different theoretical frameworks and a lack of agreement on the best way to measure these skills (Duckworth and Yeager, 2015; Napolitano et al., 2021). For this reason, the adoption of an integrative framework of skills and competencies is fundamental to increase evidence on the associations between skills, motivational and metacognitive factors, and educational outcomes (Napolitano et al., 2021). The new Social, Emotional, and Behavioral (SEB) skills framework proposed by Soto et al. (2021, 2022b) identifies core skills crucial not only for the academic domain but for everyday life situations. In this model, SEB skills are defined as people’s functional capacities to maintain social relationships, regulate emotions, manage goal-directed behaviors, and learn from experience (Napolitano et al., 2021; Soto et al., 2021; Soto et al., 2022a). Drawing from the Big Five personality traits (McCrae and Costa, 1989), SEB skills have been organized into five broad domains. The Self-Management domain (associated with Conscientiousness) refers to the capacities to effectively pursue goals and complete tasks (e.g., time management). Social Engagement skills (associated with Extraversion) include the capacities used to actively engage with other people (e.g., leading a group of people). Cooperation skills (associated with Agreeableness) are involved in maintaining positive social relationships (e.g., understanding how other people feel). Emotion Resilience skills (associated with Neuroticism) are used to regulate emotions and moods (e.g., manage anxiety). Finally, Innovation (associated with Openness) is related to those capacities used to engage with novel ideas and experiences (e.g., coming up with new ideas).

While SEB skills and Big Five personality traits are conceptually related, there are also important theoretical differences between these two constructs. While personality traits represent how an individual tends to feel, think, and behave across different situations, skills represent how an individual is capable of feeling, thinking, and behaving when the situation calls for it (Soto et al., 2022a). For example, some students may be introverted and prefer to study alone, but they also possess effective social and teamwork skills, enabling them to collaborate effectively with other students.

To assess SEB skills, a new self-report inventory was developed - the Behavioral, Emotional, and Social Skills Inventory (BESSI). The SEB skills framework and the BESSI have demonstrated optimal psychometric properties not only in their original versions (Sewell et al., 2024; Soto et al., 2022a) but also in their adaptations to other languages (Feraco et al., 2024a; Lechner et al., 2022; Postigo et al., 2024), supporting the cross-cultural validity of this framework.

The five SEB skill domains are associated with important individual characteristics such as general self-efficacy and emotional regulation strategies (Feraco et al., 2024a), as well as life outcomes, including prosocial behaviors, volunteering, social relationships, life satisfaction, and career adaptability (Breil et al., 2022; Pellegrino et al., 2025; Sewell et al., 2023; Soto et al., 2022b). With regard to academic outcomes, self-management skills have been associated to academic engagement and school grades in high school students (Soto et al., 2022b).

While it is clear that SEB skills can contribute to students’ success and well-being (Sewell et al., 2023), the mechanisms through which SEB skills influence students’ outcomes are somewhat less understood. Following previous works (Casali and Meneghetti, 2023; Feraco et al., 2022a,b), it is possible that considering SEB skills in conjunction with study-related factors (e.g., SRL and academic self-efficacy) might aid in understanding the relationship between SEB skills and students’ outcomes. A recent study (Feraco et al., 2025) in high school students has highlighted that SEB skills are positively associated with motivational and metacognitive study-related factors (e.g., SRL strategies and academic self-efficacy). Moreover, the associations between SEB skills and academic outcomes were primarily mediated by study-related factors. In other words, students with higher SEB skills tend to have higher self-efficacy and use more effective learning strategies, which can lead to better academic achievement.

To date, no study has explored the joint role of SEB skills, SRL strategies, and academic self-efficacy in explaining academic achievement and satisfaction in higher education students. The present study attempts to address this gap in the literature.

### 2.4 Research hypothesis

Drawing from previous research on the relation between soft skills, study-related factors, and student outcomes (Casali et al., 2024; Feraco et al., 2022a,b, 2025; Mega et al., 2014; Richardson et al., 2012; Soto et al., 2022b), the present study aims to build upon previous studies that considered soft skills as individual antecedents of motivational and metacognitive factors in high school (Feraco et al., 2022a,b) and university students (Casali and Meneghetti, 2023) by adopting the recent SEB skills framework (Soto et al., 2022a), which provides a comprehensive model of skills and competencies that allows distinguishing the specific contribution of each skill domain. More specifically, we hypothesized that the association between the five SEB skills domains with academic outcomes (i.e., academic achievement and satisfaction) would be explained, at least in part, by the association between SEB skills and study-related factors (i.e., academic self-efficacy and SRL).

As no previous studies had examined the relationship between SEB skill domains, academic self-efficacy, and SRL in higher education students, most of the associations were considered exploratory. However, in light of existing research (Casali et al., 2024; Feraco et al., 2025; Soto et al., 2022b), the following associations were hypothesized:

- A significant positive direct association of innovation and emotional resilience skills with academic satisfaction (Soto et al., 2022b).
- A significant indirect (through academic self-efficacy and SRL) association of self-management skills with academic achievement, as well as with academic satisfaction (Al-Abyadh and Abdel Azeem, 2022; Basileo et al., 2024; Casali et al., 2024; Feraco et al., 2022a, 2025; Lin et al., 2025).
- A significant indirect (through academic self-efficacy) association of innovation skills with academic achievement (Al-Abyadh et al., 2022; Basileo et al., 2024; Feraco et al., 2025; Lin et al., 2025).

## 3 Method

### 3.1 Participants

The sample consisted of 319 undergraduate students (average age = 22.74, *SD* = 4.68) from three countries: 128 from Spain (40.13%), 65 from Italy (20.38%), and 126 from Israel (39.50%). Of these, 255 participants were female (79.94%) and 64 participants were male (20.06%). Of the total, 111 (34.80%) participants were first-year students, 83 (26.02%) were second-year students, 100 were third-year students (31.35%), and 25 were fourth-year students (7.84%). Of the students, 161 (50.47%) reported studying education, 74 (23.20%) were in life sciences and psychology, 68 (21.32%) in hard sciences and engineering, and 15 (4.70%) in the humanities. One student did not report the field of study.

A one-way ANOVA indicated significant differences between the groups in terms of age, *F*(2, 316) = 52.19, *p* < 0.001. Bonferroni post-hoc analyses revealed that students from Israel were significantly older (*M_age_* = 25.45, *SD_age_* = 4.56) than students from Italy (*M_age_* = 22.37, *SD_age_* = 3.09) and Spain (*M_age_* = 20.25, *SD_age_* = 3.99), and students from Italy were significantly older than students from Spain. No significant differences were found in terms of sex, *F*(2, 316) = 1.02, *p* = 0.36.

### 3.2 Materials

Participants completed some introductory demographic questions about age, sex, year of course, and field of study. For all self-reported measures, responses were given on a five-point Likert scale to reduce participants’ cognitive burden and ensure higher data quality. All the instruments employed have previously been administered in this format in prior studies (e.g., Casali et al., 2024; Feraco et al., 2024a). The reliability of the questionnaires was assessed with Cronbach’s *α* and McDonald’s *ω* using the semTools package (Jorgensen et al., 2025; see Table 1). For all questionnaires, reliability indices were also calculated separately for each country to verify that reliability was adequate within each sample. Full results are provided in Supplementary Table S1.

**Table 1:** Descriptive statistics of all the variables included in the study.

Variable	Mean	SD	Skewness	Kurtosis	α	ω
Age	22.74	4.68	2.79	14.29	–	
Year of study	2.12	0.98	0.25	1.86	–	
Self-management	3.58	0.76	−0.05	2.56	0.83	0.78
Innovation	3.50	0.75	0.10	2.38	0.75	0.71
Cooperation	3.99	0.65	−0.60	3.68	0.81	0.77
Social engagement	3.44	0.78	−0.04	2.60	0.76	0.73
Emotional resilience	3.41	0.82	−0.07	2.59	0.82	0.79
Self-regulated learning	3.50	0.39	−0.13	3.29	0.74	0.70
Academic self-efficacy	3.84	0.58	−0.51	3.69	0.84	0.79
Academic satisfaction	3.51	0.82	−0.35	3.08	0.92	0.90
Achievement	3.34	0.93	−0.50	3.42	-	

#### 3.2.1 Academic satisfaction

This measure was adapted from the Multidimensional Students’ Life Satisfaction Scale (MSLSS)—Short Form—School subscale (Huebner et al., 2012). The questionnaire contained five items evaluating satisfaction with university life (e.g., “I enjoy being at the university”).

#### 3.2.2 Social, emotional, and behavioral skills

The BESSI-20 (Soto et al., 2022a; Sewell et al., 2024) is designed to measure five SEB domains: Self-management skills (e.g., “Work toward my goals”), social engagement skills (e.g., “Lead a group of people”), cooperation skills (e.g., “Understand how other people feel”), emotional resilience skills (e.g., “Calm down when I’m feeling anxious”), and innovation skills (e.g., “Understand abstract ideas”). Participants rated their ability to perform a behavior, thought, or feeling described in each item on a 5-point Likert scale (from 1 = not at all well to 5 = exceptionally well). The dependent variable is computed by averaging the scores, differentiated by the five domains.

#### 3.2.3 Academic self-efficacy questionnaire

This questionnaire (De Beni et al., 2014) consisted of five items measuring the belief that one can succeed in studying (e.g., “How do you rate your study skills?”) on a 5-point Likert scale (from 1 = poor to 5 = excellent).

#### 3.2.4 Self-regulated learning questionnaire

The short form of the Self-Regulated Learning Questionnaire (Casali et al., 2024; De Beni et al., 2014) involved 20 items assessing five SRL strategies (four items each): organization, elaboration, self-evaluation, preparing for exams, and metacognition (e.g., “I try to anticipate what kind of exam awaits me,” “When studying, I try to present the contents in my own words”). Seven items were reversed to calculate overall scores.

#### 3.2.5 Demographics and grades

The questionnaire was developed for the current study and included 14 open-ended and multiple-choice questions that referred to demographic information (e.g., sex, age, and nationality), and questions regarding participants’ studies (e.g., degree, year of study, field of study). Participants were instructed to provide their current average grades and encouraged to consult their official online academic records when reporting their grades. Grades were self-reported due to privacy regulations and the anonymity required by the ethics boards in each country. Nevertheless, prior research has shown strong correlations between self-reported and actual grades in similar contexts (e.g., Sanchez and Buddin, 2016; Sticca et al., 2017), and the anonymity of participation may have further reduced potential social desirability bias (Gnambs and Kaspar, 2015). The grades in the three countries follow different ranges: the Spanish education system uses a 10-point scale where 5 is a pass, the Italian system uses a 30-point scale where 18 is a pass, and the Israeli education system uses a 100-point scale where 60 is a pass. Grades of all three countries were converted to a 5-point scale from 1 (base level) to 5 (excellent), according to the European Credit Transfer System (ECTS) tables and grading scales, which are used to facilitate the transfer of academic results (grades) between different national assessment systems. The values used for the conversion are reported in Supplementary Table S2. To assess the validity of this conversion, we calculated Spearman’s rank correlations between the original raw grades and the converted grades within each country. The correlations were *r_s_* = 0.94 for Spain, *r_s_* = 0.92 for Italy, and *r_s_* = 0.84 for Israel, indicating that the relative ranking of students’ achievement was largely preserved.

### 3.3 Procedure

Approval to conduct the current study was obtained from the ethical review board of the authors’ institution in each country. All questionnaires were already validated in the Italian language (Feraco et al., 2024a; De Beni et al., 2014). The academic self-efficacy and the SRL questionnaires were translated from the Italian version to English and then to Spanish and Hebrew by a back-and-forth translation procedure, and the BESSI was translated from English to Spanish, and Hebrew by back-and-forth translation. Research experts in the educational and psychology fields were involved in the translation process in each country. Any controversies or doubts regarding item formulation were discussed collectively to ensure that the questionnaires were adapted to each cultural context while preserving their original meaning. We selected instruments whose items have been widely used in the literature on these topics across different national contexts to minimize the risk of cultural bias. For example, although recent, the BESSI has already been validated in multiple cultural contexts, including Italy (Feraco et al., 2024a) and Spain (Postigo et al., 2024). The MSLSS has also demonstrated cross-cultural validity in previous studies (e.g., Jiang et al., 2021). The questionnaires on academic self-efficacy and SRL strategies align with common operationalizations of these constructs that are widely adopted in the international literature (Fong et al., 2021; Richardson et al., 2012). Taken together, these steps increase the likelihood of the instruments being appropriate for use in all three participating countries.

The study is the product of a long academic acquaintance that gave rise to this research collaboration. The study was conducted in the second semester of the academic year 2023 (from March to June). Each co-researcher collected the data in their own country. The students were recruited primarily through an online survey circulated via social media and academic digital platforms. The first page of the survey contained the informed consent form, and the beginning of the survey constituted the provision of consent. It was made clear to participants that participation in the study was voluntary and anonymous and that the study adhered to all ethical guidelines. Questionnaires were administered in the order presented above.

### 3.4 Data analysis

All analyses were conducted using the lavaan package (Rosseel, 2012) in R (R Core Team, 2024). Preliminary analyses included descriptive statistics and Pearson correlations to assess bivariate associations among all the variables.

We then fitted a saturated path model in which SEB skills predicted self-regulated learning and academic self-efficacy, and in which SEB skills, self-regulated learning, and academic self-efficacy jointly predicted academic satisfaction and achievement. Sex, age, year of study, and nationality (dummy variable) were included in the model as control variables, to account for potential sample differences and ensure they did not confound the associations tested in the path analysis. All the indirect associations between SEB skills and student outcomes (through self-regulated learning and academic self-efficacy) were also calculated. We used maximum likelihood estimation with 5,000 bootstrap samples to obtain robust standard errors (Beaujean, 2014). Effect sizes were quantified as standardized path coefficients (*β*) for direct and indirect associations, with 95% confidence intervals, and as R^2^ values for each endogenous variable.

## 4 Results

### 4.1 Preliminary analyses

Descriptive statistics are reported in Table 1. To examine the bivariate associations between all variables, Pearson correlation analyses were conducted. As presented in Table 2, significant positive relationships were found between all research variables.

**Table 2:** Correlation matrix (Pearson’s r) with all the variables involved in the study.

Variable	1	2	3	4	5	6	7	8	9	10
1. Age										
2. Year of study	0.37 ***									
3. Self-management	0.24***	0.07								
4. Innovation	0.30***	0.15**	0.49***							
5. Cooperation	0.23***	0.07	0.41***	0.47***						
6. Social Engagement	0.22***	0.10	0.50***	0.52***	0.61***					
7. Emotional Resilience	0.23***	0.08	0.46***	0.55***	0.61***	0.47***				
8. SRL	−0.02	0.00	0.43***	0.32***	0.22***	0.34***	0.23***			
9. Self-efficacy	0.14*	0.05	0.60***	0.40***	0.41***	0.51***	0.39***	0.47***		
10. AS	0.06	−0.12*	0.32***	0.33***	0.29***	0.34***	0.27***	0.33***	0.42***	
11. Achievement	0.25***	0.15**	0.41***	0.24***	0.30***	0.33***	0.26***	0.28***	0.51***	0.14*

SRL, Self-regulated learning; AS, Academic satisfaction. *p < 0.05, **p < 0.01, ***p < 0.001.

### 4.2 Path analysis: direct associations

To assess the direct and indirect (through self-regulated learning and academic self-efficacy) associations between SEB skills (independent variables) and academic achievement and satisfaction (dependent variables), a path analysis was conducted. The model explained 43% of the variance for academic self-efficacy, 31% for SRL strategies, 32% for academic satisfaction, and 41% for grades. Standardized residual variances for all endogenous variables are reported in Supplementary Table S3.

**Figure 1:**
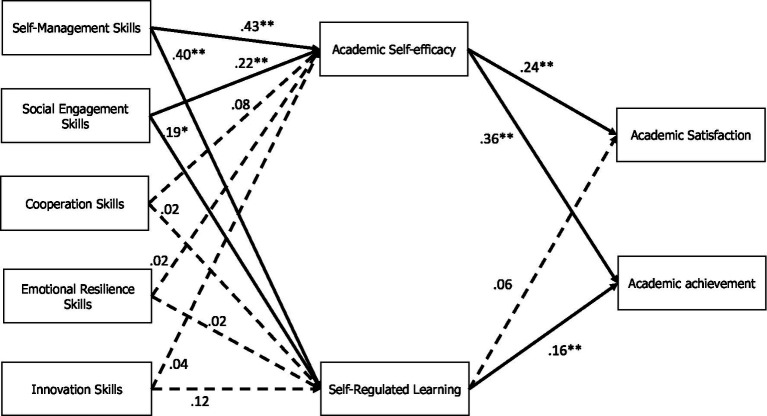
Fitted path analysis illustrating the relationship between behavioral, emotional, and social skills and academic achievements, mediated by academic self-efficacy and self-regulated learning. For the sake of readability, in this figure direct associations between SEB skills and academic outcomes (achievement and satisfaction) and residual variances are not reported.

Results of the fitted model are detailed in Figure 1 and Tables 3, 4. Regarding direct associations, results showed that self-management skills were positively associated with SRL (*β* = 0.40, *p* < 0.001) and academic self-efficacy (*β* = 0.43, *p* < 0.001). Moreover, social engagement skills were positively associated with SRL strategies (*β* = 0.19, *p* < 0.01) and academic self-efficacy (*β* = 0.22, *p* < 0.001). However, no significant direct association was found between SEB skills and academic satisfaction and achievement. Academic self-efficacy was positively associated with both academic satisfaction (*β* = 0.24, *p* < 0.001) and achievement (*β* = 0.36, *p* < 0.001). Finally, SRL strategies were positively associated with academic achievement (*β* = 0.16, *p* < 0.001).

**Table 3:** Results of the path analysis model: direct associations.

Independent variable		Dependent variable	β	*p*-value	95% CI
Sex	→	ASE	0.00	0.94	[−0.10; 0.09]
	→	SRL	−0.01	0.90	[−0.11; 0.10]
	→	AS	0.02	0.73	[−0.08; 0.11]
	→	Achievement	−0.09	0.06	[−0.19; 0.01]
Age	→	ASE	−0.05	0.35	[−0.14; 0.05]
	→	SRL	−0.06	0.34	[−0.20; 0.07]
	→	AS	0.09	0.11	[−0.02; 0.20]
	→	Achievement	0.03	0.63	[−0.10; 0.17]
Year of study	→	ASE	0.03	0.61	[−0.08; 0.13]
	→	SRL	0.05	0.44	[−0.07; 0.17]
	→	AS	−0.07	0.21	[−0.17; 0.04]
	→	Achievement	0.05	0.36	[−0.05; 0.15]
Italy (dummy variable)	→	ASE	−0.04	0.56	[−0.17; 0.09]
	→	SRL	0.23	< 0.001	[0.11; 0.36]
	→	AS	0.04	0.49	[−0.08; 0.16]
	→	Achievement	−0.32	< 0.001	[−0.44; −0.20]
Spain (dummy variable)	→	ASE	0.03	0.62	[−0.09; 0.16]
	→	SRL	0.34	< 0.001	[0.21; 0.46]
	→	AS	0.33	< 0.001	[0.21; 0.46]
	→	Achievement	−0.40	< 0.001	[−0.52; −0.27]
Self-management	→	ASE	0.43	< 0.001	[0.33; 0.54]
	→	SRL	0.40	< 0.001	[0.29; 0.50]
	→	AS	0.07	0.35	[−0.07; 0.20]
	→	Achievement	0.02	0.71	[−0.09; 0.13]
Innovation	→	ASE	0.04	0.48	[−0.08; 0.16]
	→	SRL	0.12	0.06	[−0.01; 0.24]
	→	AS	0.15	0.05	[−0.00; 0.29]
	→	Achievement	−0.04	0.51	[−0.16; 0.08]
Cooperation	→	ASE	0.08	0.27	[−0.06; 0.21]
	→	SRL	0.02	0.78	[−0.12; 0.16]
	→	AS	0.10	0.14	[−0.03; 0.23]
	→	Achievement	−0.02	0.78	[−0.14; 0.10]
Social engagement	→	ASE	0.22	< 0.001	[0.09; 0.35]
	→	SRL	0.19	0.001	[0.06; 0.31]
	→	AS	0.09	0.23	[−0.06; 0.24]
	→	Achievement	−0.02	0.78	[−0.15; 0.12]
Emotional resilience	→	ASE	0.02	0.74	[−0.11; 0.15]
	→	SRL	0.02	0.77	[−0.12; 0.16]
	→	AS	0.00	0.95	[−0.12; 0.13]
	→	Achievement	−0.05	0.41	[−0.18; 0.07]
Academic self-efficacy	→	AS	0.24	< 0.001	[0.11; 0.38]
	→	Achievement	0.36	< 0.001	[0.26; 0.46]
Self-regulated learning	→	AS	0.06	0.29	[−0.05; 0.17]
	→	Achievement	0.16	< 0.001	[0.07; 0.26]

ASE, academic self-efficacy; SRL, SRL strategies; AS, academic satisfaction; β, standardized coefficient; CI, confidence interval.

**Table 4:** Results of the path analysis model: indirect associations.

Independent variable	Mediator	Dependent variable	β	*p*-value	95% CI
Self-management →	ASE →	AS	0.11	< 0.001	[0.04; 0.17]
	ASE →	Achievement	0.16	< 0.001	[0.10; 0.21]
	SRL →	AS	0.02	0.29	[−0.02; 0.07]
	SRL →	Achievement	0.06	0.003	[0.02; 0.11]
Innovation →	ASE →	AS	0.01	0.48	[−0.02; 0.04]
	ASE →	Achievement	0.02	0.48	[−0.03; 0.06]
	SRL →	AS	0.01	0.34	[−0.01; 0.02]
	SRL →	Achievement	0.02	0.12	[−0.00; 0.04]
Cooperation →	ASE →	AS	0.02	0.29	[−0.02; 0.05]
	ASE →	Achievement	0.03	0.27	[−0.02; 0.07]
	SRL →	AS	0.00	0.79	[−0.01; 0.01]
	SRL →	Achievement	0.00	0.78	[−0.02; 0.03]
Social Engagement →	ASE →	AS	0.05	0.02	[0.01; 0.10]
	ASE →	Achievement	0.08	0.004	[0.03; 0.13]
	SRL →	AS	0.01	0.33	[−0.01; 0.03]
	SRL →	Achievement	0.03	0.03	[0.00; 0.06]
Emotional Resilience →	ASE →	AS	0.01	0.74	[−0.03; 0.04]
	ASE →	Achievement	0.01	0.74	[−0.04; 0.05]
	SRL →	AS	0.00	0.78	[−0.01; 0.01]
	SRL →	Achievement	0.00	0.77	[−0.02; 0.03]

ASE, academic self-efficacy; SRL, SRL strategies; AS, academic satisfaction; β, standardized coefficient; CI, confidence interval.

### 4.3 Path analysis: indirect associations

As for the indirect associations, results showed that self-management and social engagement skills were positively associated with academic achievement through the mediation of SRL (self-management: *β* = 0.06, *p* < 0.01; social engagement: *β* = 0.03, *p* < 0.05) and academic self-efficacy (self-management: *β* = 0.06, *p* < 0.01; social engagement: *β* = 0.03, *p* < 0.05), and with academic satisfaction through the mediation of academic self-efficacy.

## 5 Discussion

Research on higher education students has attempted to identify key individual factors associated with academic achievement and satisfaction (Casali et al., 2024; Richardson et al., 2012). Recently, researchers have identified soft skills as individual competencies that could support students throughout their academic journey (Casali and Meneghetti, 2023). The present study proposed a comprehensive model in which five SEB domains (self-management, social engagement, cooperation, emotional resilience, and innovation; Soto et al., 2021) were associated with achievement and academic satisfaction through the mediation of two study-related factors - academic self-efficacy and SRL (García-Ros et al., 2023; Mega et al., 2014; Theobald, 2021). The study was conducted in a sample of higher education students from Spain, Italy, and Israel.

Overall, the study’s findings support previous literature regarding the influence of study-related factors on academic success and satisfaction (Casali and Meneghetti, 2023; Diseth et al., 2012; Richardson et al., 2012). In particular, self-management and social engagement skills were indirectly associated with academic satisfaction through academic self-efficacy and with academic achievement through self-efficacy and SRL. These findings highlight the importance of considering the interplay between SEB skills and study-related factors in promoting student success and satisfaction.

### 5.1 Direct association between SEB skills, academic self-efficacy, SRL, and academic outcomes

As a first step, the direct associations of SEB skills, academic self-efficacy, and SRL on academic achievement and satisfaction were tested. Our results indicated that SEB skills were not directly associated with academic achievement and satisfaction. These findings are in line with previous research on soft skills, which highlighted that skills may not have a direct impact on educational outcomes, but their effect may be mediated by other individual factors, such as motivational and metacognitive study-related factors (Casali and Meneghetti, 2023; Feraco et al., 2022a,b).

Although our hypothesis predicting a significant positive direct association between innovation skills, emotional resilience, and academic satisfaction was not supported by the path analysis model, the bivariate correlations indicated that these skills were positively related to this outcome, consistent with previous research (Soto et al., 2022b). This finding suggests that innovation and emotional resilience remain relevant, although other domains (i.e., self-management and social engagement) may play a larger role in explaining variability in academic satisfaction.

A significant relationship between academic self-efficacy and academic achievement and satisfaction was found (Casali et al., 2024; Høigaard et al., 2015; Lee et al., 2022). Students who perceived themselves as more capable of meeting academic demands also reported higher levels of academic satisfaction and better achievement. Academic self-efficacy is one of the most important intraindividual antecedents of academic outcomes in higher education (Bandura, 1982, 2012; Casali et al., 2024; Richardson et al., 2012) and one of the most powerful motivational resources that drive individuals to engage, persevere, and accomplish goals in various domains (Lee et al., 2022). SRL was significantly associated only with achievement: students who reported using functional SRL strategies more often also reported higher grades (Casali et al., 2022; Richardson et al., 2012).

### 5.2 SEB skills, study-related factors, and academic outcomes: indirect associations

In line with previous studies (Casali and Meneghetti, 2023; Feraco et al., 2022a,b), we expected that the associations between SEB skills and academic outcomes would be partly explained by the associations between SEB skills and study-related factors. The results confirmed an indirect association between self-management and social engagement skills with academic satisfaction through academic self-efficacy: higher levels of reported self-management and social engagement skills were positively related to higher academic self-efficacy, which in turn, was related to higher academic satisfaction. Self-management skills include the abilities to set goals, manage time and tasks, and be persistent in achieving goals (Napolitano et al., 2021). Thus, students with higher self-management skills may perceive themselves as more capable of organizing their academic activities, managing daily study routines, and achieving their desired academic goals (Mega et al., 2014; Theobald, 2021). In addition, social engagement skills include the ability to initiate conversations with others and express personal opinions. Since active engagement with peers and instructors is an integral part of the college experience (Reeve et al., 2022), students who perceive themselves as more socially proficient may also perceive themselves as more academically competent. The development of social engagement skills may enhance students’ sense of academic competence, as they are better capable of seeking assistance from their peers when required (Dueñas et al., 2021), expressing opinions during lessons, asking questions, and expressing their preferences regarding learning conditions with the academic staff (Reeve et al., 2022).

Finally, self-management and social engagement skills were indirectly associated with academic achievement through academic self-efficacy and SRL. Students who reported higher self-management and social engagement skills also reported higher academic self-efficacy and the use of more functional SRL strategies. These students may use their general self-management skills to organize their study routines and identify the most functional learning strategies (Feraco et al., 2025). Moreover, they may benefit from their peer support in navigating study-related challenges (Napolitano et al., 2021). This, in turn, can help students achieve better grades (Feraco et al., 2025; Mega et al., 2014; Wang et al., 2019).

Our hypothesis predicting an indirect association between innovation skills and academic achievement via academic self-efficacy was not supported by the path analysis model. Nonetheless, significant correlations emerged both between innovation skills and self-efficacy, and between self-efficacy and achievement, aligning with previous research (Basileo et al., 2024; Feraco et al., 2025). This pattern suggests that although innovation skills relate to self-efficacy and achievement, other SEB skill domains may be more central in accounting for variability in these outcomes.

Taken together, our findings confirm that self-management and social engagement skill domains may represent domain-general individual capacities that can support students academically (Casali and Meneghetti, 2023; Feraco et al., 2022a, 2025; Wang et al., 2019). They suggest that when working with higher education students, it may be important to consider not only study-related factors but also general individual characteristics that could contribute to their success at university (Casali et al., 2024).

### 5.3 Implications for theory and practice

The results of the study have several theoretical and practical implications. From a theoretical perspective, they underline that students’ success in higher education is not only associated with motivation and metacognitive factors but also with domain-general SEB skills, that are important in the academic context and beyond (Casali and Meneghetti, 2023; Soto et al., 2022b). In addition, the recent SEB skills framework (Soto et al., 2022a) appears to be a solid theoretical model for exploring the specific contribution of different skill domains, as each may have different associations with study-related factors and educational outcomes.

The present study also holds important practical implications. Typically, higher education institutions organize activities designed to enhance only study-related skills, such as SRL strategies (Pellegrino et al., 2023). However, the present study suggests that a more comprehensive approach to learning processes and students’ well-being may include the promotion of a wider range of SEB skills. Promoting these skills aims to equip students with the competencies necessary to adapt to the academic environment and navigate potential academic challenges. At the same time, these skills are considered fundamental in various careers, so it should be an important goal of higher education institutions to prepare students for the transition to work by fostering these competencies (Feraco et al., 2022a; Napolitano et al., 2021).

### 5.4 Limitations and future directions

The current study is subject to certain limitations. Primarily, the study relied on student self-report of their grades, which were not directly verified. Future studies could verify grades through official academic records to further strengthen the robustness of the findings.

Secondly, due to the relatively small sample sizes in each country, it was not feasible to test the invariance of the path model, the measurement invariance of the questionnaires, or the potential moderating effects of nationality. To mitigate this limitation, nationality was controlled for in the path analysis model. Future research should examine both measurement and structural invariance, and compare the role of SEB skills in academic achievement and satisfaction across different cultural contexts to identify possible patterns of relations specific to each context.

Another limitation is that our study employed a cross-sectional design, which precludes the possibility of inferring causal relationships between the variables. Future research should use longitudinal or experimental designs to collect more evidence on the causal associations between these variables.

Our sample included a majority of female participants and undergraduate students from the field of education sciences, life sciences, and psychology, a pattern also observed in studies using similar recruitment approaches (e.g., Casali and Meneghetti, 2023). In addition, there were age differences across the three nations. To enhance the generalizability of our findings, future research should aim for a more diversified sample, for example by employing stratified sampling strategies.

In our study, emotional resilience, innovation, and cooperation skills showed positive correlations with academic satisfaction and achievement but were not significant predictors in the path analysis model. This discrepancy may reflect the stronger role of self-management and social engagement domains, which likely account for a larger share of the variability in satisfaction and achievement. Nonetheless, emotional resilience, innovation, and cooperation skills may still be relevant for other important academic outcomes, such as coping with stress and challenges (Fiorilli et al., 2022) and positive participation in group activities (Poort et al., 2022). Future research should therefore consider a broader range of outcomes to clarify the role of these skills in higher education.

Finally, given the pivotal role that SEB skills may play in supporting higher education students, it would be relevant to investigate their importance for students facing specific learning challenges, for example, students with specific learning disorders and attention deficit hyperactivity disorder (Casali et al., 2024).

## 6 Conclusion

The present study proposed a comprehensive model in which five SEB skill domains are associated with achievement and academic satisfaction directly and through the mediation of two study-related factors – academic self-efficacy and SRL. We hypothesized an indirect association of self-management skills with academic achievement as satisfaction trough self-efficacy and SRL. Moreover, we expected a significant indirect association of innovation skills with academic achievement. Finally, we expected positive associations between innovation and emotional resilience skills with academic satisfaction. The results confirmed our hypothesis that self-management would be positively associated with academic achievement through the mediation of SRL and academic self-efficacy, and with academic satisfaction through the mediation of self-efficacy. Additionally, social engagement skills showed a similar pattern of mediated associations. However, no significant direct associations were found between SEB skills and either academic satisfaction or academic achievement. The results suggest that students who believe they possess some social, emotional, and behavioral skills that can be beneficial during their studies (such as self-management skills and social engagement), perceive themselves as more competent and self-regulated learners. This perception may lead to better academic results and greater satisfaction with their academic pursuits.

## Data Availability

The raw data supporting the conclusions of this article will be made available by the authors, without undue reservation.
