# Supplementary material for: Undergraduates’ achievement and satisfaction: the role of study-related factors and soft skills
Source: Front Psychol. 2025 Oct 8;16:1653072. doi: 10.3389/fpsyg.2025.1653072 (PMC12540472; doi:10.3389/fpsyg.2025.1653072)
Supplement: Supplementary file 1 [file Table_1.docx]

Supplementary Materials

# Table S1. Internal consistency (Cronbach’s α and McDonald's ω) by country

|  | **α** | | | **ω** | | |
| --- | --- | --- | --- | --- | --- | --- |
|  | **Spain** | **Italy** | **Israel** | **Spain** | **Italy** | **Israel** |
| ***Academic Satisfaction*** | .90 | .90 | .93 | .87 | .87 | .91 |
| ***SEB skills:*** |  |  |  |  |  |  |
| Self-management skills | .82 | .79 | .83 | .78 | .73 | .77 |
| Social engagement skills | .72 | .68 | .75 | .68 | .68 | .71 |
| Cooperation skills | .78 | .77 | .76 | .73 | .71 | .70 |
| Emotional resilience skills | .73 | .73 | .83 | .72 | .70 | .79 |
| Innovation skills | .74 | .73 | .75 | .70 | .68 | .70 |
| ***Academic Self-Efficacy*** | .75 | .82 | .87 | .70 | .75 | .85 |
| ***Self-Regulated Learning*** | .76 | .76 | .76 | .74 | .80 | .72 |

# Table S2. Conversion Table for Academic Grades

| **ISRAEL** | **ITALY** | **SPAIN** | **Verbal score** | **Numerical Value** |
| --- | --- | --- | --- | --- |
| 95-100 | >30 | >9 | Excellent | 5 |
| 85-94 | 28-29 | 8-8.9 | Very good | 4 |
| 75-84 | 25-27 | 7-7.9 | Good | 3 |
| 65-74 | 20-24 | 6-6.9 | Satisfactory | 2 |
| 60-64 | 18-19 | 5-5.9 | Base | 1 |
| 0-59 | <18 | <5 | Fail | 0 |

# Table S3. Standardized residual variances for endogenous variables in the path analysis model.

| **Endogenous variable** | **Std. Var.** | **SE** | **p-value** | **95% CI** |
| --- | --- | --- | --- | --- |
| Academic Self-Efficacy | .57 | .04 | < .001 | [.49; .66] |
| Self-Regulated Learning | .69 | .04 | < .001 | [.60; .78] |
| Academic Satisfaction | .68 | .05 | < .001 | [.59; .78] |
| Achievement | .59 | .05 | < .001 | [.49; .68] |
